# Potential Role for Stem Cell Regenerative Therapy as a Treatment for Degenerative Disc Disease and Low Back Pain: A Systematic Review

**DOI:** 10.3390/ijms24108893

**Published:** 2023-05-17

**Authors:** Khadija H. Soufi, Jose A. Castillo, Freddie Y. Rogdriguez, Charles J. DeMesa, Julius O. Ebinu

**Affiliations:** 1 Department of Neurological Surgery, University of California Davis, Sacramento, CA 95817, USA; 2 Department of Anesthesia and Pain Medicine, University of California Davis, Sacramento, CA 95817, USA

**Keywords:** stem cells, lower back pain, regenerative medicine, degenerative disc, discogenic

## Abstract

Back pain is the single leading cause of disability worldwide. Despite the prevalence and morbidity of lower back pain, we still lack a gold-standard treatment that restores the physiological function of degenerated intervertebral discs. Recently, stem cells have emerged as a promising strategy for regenerative therapy for degenerative disc disease. In this study, we review the etiology, pathogenesis, and developing treatment strategies for disc degeneration in low back pain with a focus on regenerative stem cell therapies. A systematic search of PubMed/MEDLINE/Embase/Clinical Trials.gov databases was conducted for all human subject abstracts or studies. There was a total of 10 abstracts and 11 clinical studies (1 RCT) that met the inclusion criteria. The molecular mechanism, approach, and progress of the different stem cell strategies in all studies are discussed, including allogenic bone marrow, allogenic discogenic cells, autologous bone marrow, adipose mesenchymal stem cells (MSCs), human umbilical cord MSC, adult juvenile chondrocytes, autologous disc derived chondrocytes, and withdrawn studies. Clinical success with animal model studies is promising; however, the clinical outcomes of stem cell regenerative therapy remain poorly understood. In this systematic review, we found no evidence to support its use in humans. Further studies on efficacy, safety, and optimal patient selection will establish whether this becomes a viable, non-invasive therapeutic option for back pain.

## 1. Introduction

Back pain is the single leading cause of disability worldwide affecting people of all ages and socioeconomic statuses [1,2,3,4,5]. In North America, it is one of the most common reasons for missed days at places of employment, and it is the third most common cause for visits to a physician’s office, behind only dermatological and osteoarthritic/joint complaints [3]. At least 80% of Americans will experience low back pain (LBP) during their lifetime. The National Institute of Neurological Disorders and Stroke (NINDS) rates back pain as the most common cause of job-related disability [6]. Consequently, LBP costs Americans over USD 50 billion in healthcare costs every year and even more in lost income and revenue due to decreased productivity [3,4].

The cause of lower back pain is complex and multifactorial, and often the specific source of the pain cannot be identified. For a significant portion of patients with back pain, the symptoms are short-lived and self-limiting in nature, with occasional recurrent episodes. A small percentage of patients develop chronic and disabling LBP. Organic causes of back pain, such as infection, malignancy, inflammation, or trauma, are a rarity since most back pain is mechanical in nature.

While diagnostic tests may not always accurately identify the precise source of LBP, several innervated structures of the spinal anatomy have been implicated as pain generators. It is becoming increasingly evident that one of the foremost causes of LBP is intervertebral disc degeneration [7]. Degenerative discs are associated with axial LBP and/or radiculopathy secondary to lumbar spondylosis. In addition, degenerative discs have also been implicated in axial neck pain and myelopathy in the cervical spine.

Despite the prevalence and morbidity associated with LBP in the setting of degenerative disc disease, there is no gold standard for the treatment of LBP, which restores the integrity and normal physiological function of degenerated intervertebral discs. Considering this, we reviewed some of the developing and new treatment strategies for low back pain that target disc degeneration in patients with severe back pain related to degenerative disc disease.

### 1.1. Structure and Anatomy of Intervertebral Discs

**Figure 1:**
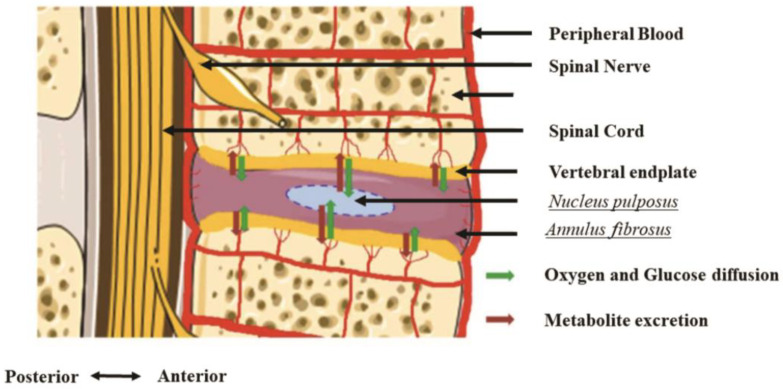
Healthy intervertebral disc (IVD) local vascularization, innervation, and internal organization. Note the position of the two vertebral endplates. Furthermore, observe that the oxygen and glucose supply, as well as metabolite excretion, occur by diffusion through the vertebral endplates. This property induces specific conditions of osmolarity and pH, leading the IVD resident cells to adapt their metabolism. In the process of degenerative changes, some studies suggest that native cells in the IVD and cartilage are able to tolerate acidic conditions, which is believed to be due to their expression of specific acid-sensing ion channels [12,13].

Intervertebral discs are fibrocartilaginous in nature, contributing to their role in providing mobility and maintaining the integrity of the spine (Figure 1). There are three main structural components in intervertebral discs: (1) nucleus pulposus (NP), (2) annulus fibrosis (AF), and (3) cartilaginous endplates (EPs) [8,9]. The NP is the inner core of the disc and consists of a gelatinous extracellular matrix (ECM), which is made up of predominantly proteoglycans produced by large notochordal cells and type II collagen [8,10]. This NP inner core is enclosed by an organized collagenous lamella comprising fibrous concentric layers of type I collagen—the AF [8,10]. Lastly, the cartilaginous EPs provide the disc connection to its neighboring vertebral bodies by separating thin layers of hyaline cartilage that bind the disc to the adjacent bony vertebrae [8,10].

As a structural unit, the intervertebral disc, the NP, AF, and EPs are interconnected to form a crucial part of the motion segment of the spine. The discs function as shock absorbers and provide resistance to both the tensile and torsional forces. The matrix of the NP is approximately 80% water, which helps it resist compressive forces and provide flexibility to the spine [11]. The NP and the AF work together to absorb shock in the spinal column, and the AF also helps to provide tensile strength [11]. The EPs are uniquely designed to mediate disc and vertebral health. Their porosity facilitates chemical transport, while their strength resists a mechanical collapse between the disc and vertebrae.

Intervertebral discs are also the largest avascular tissue in the body as well as aneural. To maintain the viability of the NP cells, the intervertebral discs primarily attain their nutrients and metabolites by diffusion from vessels in the cartilaginous EP and outer AF (Figure 1) [8,10].

### 1.2. Pathophysiology of Degenerative Disc Disease and Back Pain

**Figure 2:**
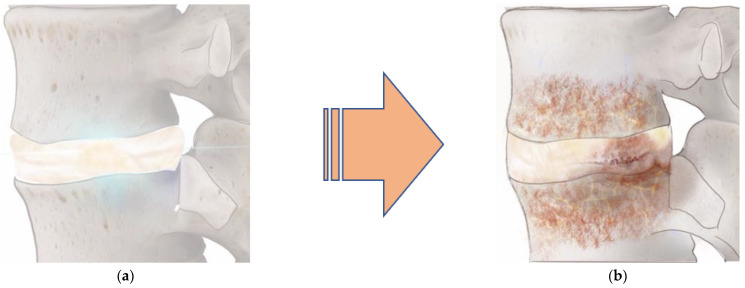
Factors influencing IVD microenvironment. Over time, intervertebral discs lose their integrity and structure (**a**) and undergo a degenerative process. Factors contributing to disc degeneration include hypoxia, low nutrient conditions, hyperosmolarity (excess leads to catabolism), acidity (pH), and inflammation. These factors lead to a decline in the balance between extracellular matrix production and apoptosis, resulting in a degenerated intervertebral disc (**b**).

Both the integrity and the structure of the discs change with age (Figure 2). Degenerative disc changes are associated with cell-mediated responses to structural failure. These age-related changes have been demonstrated to cause an increase in the size and number of tissue fissures, granular debris, and neovascularization from the outer aspect of the annulus inwards [12,13]. Thus, as one ages the NP changes from a gelatinous structure to a more fibrous one because its proteoglycan content and hydration diminish. This structural change leads to the development of clefts that extend through the AF and result in a progressive decrease in the NP size and overall disc height [12,13]. Additional age-related changes cause ossification and thinning of the endplates, development of microfractures in the adjacent subchondral bone structure, bone sclerosis, and significant reduction in the vasculature of the cartilaginous endplate [12,13]. The reduced vascular supply in the endplates contributes to further decreased nutrient supply, leading to hypoxia, accumulation of cellular debris and waste products, and an acidic environment that impairs disc cell function in synthesizing and supporting the ECM [12,14,15,16]. Ultimately, these changes in the ECM lead to the degeneration of the disc.

While aging can contribute to disc disease, disc degeneration may also occur independent of the aging process. It is difficult to clearly differentiate the physiological process of disc aging from that of disc degeneration. As such, the main distinguishing factor in disc degeneration is the inherent structural and functional failure of the disc secondary to impaired cellular processes and extracellular matrix changes [12,13]. In the early phase of disc degeneration, clefts and fissures occur in the nucleus and inner annulus causing chondrocyte-like cells in the inner annulus to proliferate and produce a matrix around the structural defect [12,13]. Over time, the structural defect progresses due to its inability to withstand the compressive forces on the spine. As the degeneration advances, the tears extend into the outer annulus and become filled with granular material [12,14,15,16]. The late stage of disc degeneration is marked by an increase in collagen content in the disc, eventually leading to the formation of fibrotic material. The structural failure of the disc in combination with the physiological process of aging, accelerated by factors such as genetics, obesity, nutrition, smoking, inflammation, as well as catabolic cytokines and proteases, leads to an imbalance between the anabolic and catabolic processes involved in the maintenance of the ECM [12,17,18,19,20].

Intervertebral disc degeneration is a major cause of neck and back pain and a major cause of disability [12,13]. The pathophysiology of discogenic back pain involves an imbalance in the anabolic and catabolic environments of the extracellular matrix in favor of catabolism [12,16,21,22]. The resultant alteration in disc height affects the biomechanics of the involved spinal segment, which often results in segmental instability [23]. Consequently, other supportive spinal structures are often affected, which culminates in back pain associated with degenerative spondylosis. In addition, the proinflammatory state that is created in the outer AF tears can stimulate nociceptive nerve endings there [24,25]. Recent studies have identified an association between this increased nociceptive nerve ingrowth and granulation tissue in the AF with the pain from disc degeneration [26,27,28,29]. This finding provides a direct link between low back pain and disc degeneration and a leading premise in the pathophysiology of discogenic back pain.

### 1.3. Stem Cell Regenerative Therapies and Discogenic Back Pain

Current approaches to treat discogenic back pain primarily target symptomatic relief through either conservative or invasive methods [30,31,32]. Conventional therapies primarily target symptom control and fail to counter disc degeneration or restore disc function and structure. Our understanding of the pathophysiology of discogenic back pain has generated considerable interest in cell-based approaches to counteract the degenerative process, with hopes of restoring normal cellular activity and ECMs in intervertebral discs [33,34]. While various cell types have been used to provide regenerative therapy in patients with discogenic back pain, stem cells have presented a promising option for this strategy.

Stem cells are unspecialized cells with the ability to self-renew and differentiate into various cell types [35,36]. Recent treatments have primarily explored the use of notochordal cells, chondrocyte-like and other nucleus pulposus-derived cells, and mesenchymal stromal cells to treat discogenic back pain with promising preliminary findings [37,38,39,40,41]. Irrespective of the stem cell treatment strategy, the ideal modality requires being able to conserve the physiologic function and environment of discs, while reversing the degenerative cascade that ensues as a function of age and other contributing factors. The key factor making stem cell treatment particularly attractive involves their potential to repopulate the disc with viable cells and their ability to restore damaged tissue and enhance tissue regeneration by modulating the inflammatory response [29,33,34,42,43,44,45].

Mesenchymal stem cells (MSCs) have proven particularly attractive for regenerative cell therapy for degenerative disc disease. Given their capability of differentiating into various connective tissues cells and being readily obtained from several sources, including adipose tissue, muscles, umbilical cord blood, bone marrow, and dermis, MSCs could potentially provide the ideal treatment for degenerative disc disease and discogenic back pain [29,46,47]. In this review, we focus on MSCs and their role in treating discogenic LBP.

## 2. Methods

**Figure 3:**
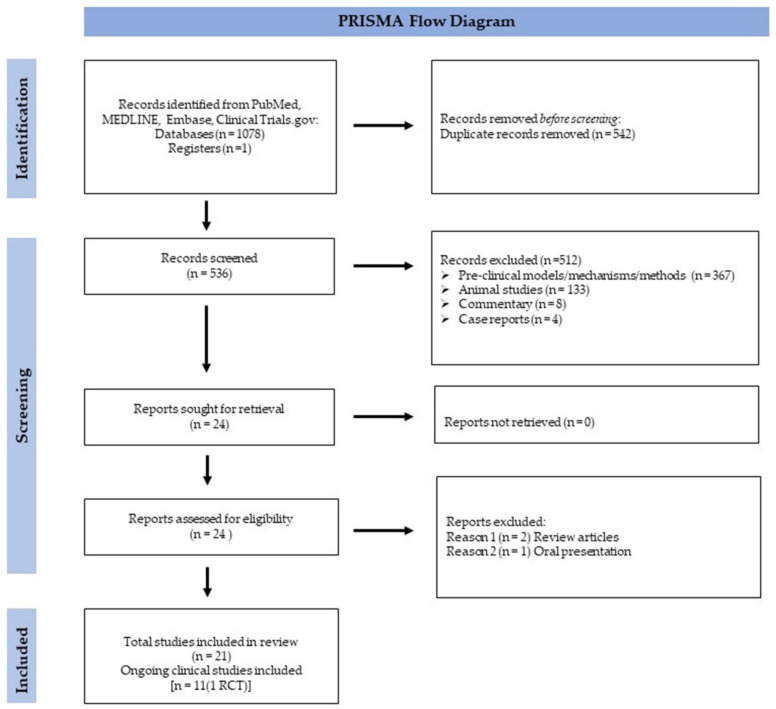
Preferred Reporting Items for Systematic Reviews and Meta-Analyses (PRISMA) diagram for the systematic review. Abbreviations: RCT (randomized control trial).

A systematic search of PubMed/MEDLINE was conducted for literature published until 17 October 2022 in the EMBASE and Clinical Tri-als.gov databases. Only studies in human subjects with abstracts written in English were considered for inclusion, while no other search limitations were included (Figure 3). We sought to identify stem cell use versus conservative care, other disc-preserving injection treatments, and surgical care in patients with lumbar degenerative disc disease (DDD), or disc herniation. The search strategy included the use of controlled vocabulary (MeSH terms) and search terms, such as a vertebra, low back pain, intervertebral disc degeneration, stem cells, mesenchymal stem cells, cell therapy, progenitor cells, stromal cells, bone marrow (BM) aspirate, BM concentrate, adipose tissue-derived cells, lipoaspirate, stromal vascular fraction, chondrocytes, and nucleus pulposus cell. A summary of findings is reported and organized by findings and data treatment type (Table 1). Clinical trials by type of stem cell therapy are listed in (Table 2).

## 3. Results and Discussion

### 3.1. Literature Search Results

The initial search resulted in 1078 articles. After duplicate articles were removed (*n* = 542), 536 unique articles were identified. After the initial screening, 512 articles did not meet the inclusion criteria, most being related to preclinical models/mechanisms/methods, involving animal studies, commentary, or case reports (Figure 3). The remaining 24 articles were reviewed, of which 2 were excluded as review articles, and 1 article as an oral presentation. A total of 21 articles met our final inclusion criteria for PRISMA. Among the final articles, there was a total of 10 abstracts and 11 clinical studies (1 RCT).

### 3.2. Patient Characteristics and Demographics

The included studies described a total of 119 patients. The mean age was 38 (ranging from 18 to 64). Gender was documented in all reports with a total number of 71 males and 48 females included in all articles. There was an average of 15 months of follow-up (ranging from 12 to 72). Validated patients reported outcomes comprising the Oswestry Disability Index (ODI) and visual analog scale for pain (VAS), except for one study, which reported the Japanese Orthopedic Association score (JOA), Functional Rating Index (FRI), and numeric pain scale (NPS). ODI is a validated outcome measure from 0 to 100, with 0 representing functional independence and 100 being severely disabled. The VAS is a pain-reported outcome scale with 0 being no pain and 10 being the worst pain possible. JOA is reported on a scale of 0 (severely disabled) to 17 (no disability). FRI is reported from 0 (functionally independent) to 100 (severely disabled). NPS has a pain scale with a range from 0 (no pain) to 10 (worst possible pain).

### 3.3. Clinical Studies: Potential Role of Stem Cell Therapy for Degenerative Disc Disease and Low Back Pain

In a prospective cohort study of 10 patients treated with autologous MSCs (10 **×** 10^6^ cells/disc, isolated from subcutaneous adipose tissue) for low back pain caused by degenerative disc disease (DDD), they reported reductions of 68% and 62.4% in VAS and ODI, respectively, at 6 months [50]. These results remained stable after 12 months with no significant change in disc height but radiographic signs of increased water content on the MRI. A total of 60% of the patients in this study were treated at the L5–S1 disc level and the results were independent of the level injected.

In a separate study by Mochida et al., autologous bone marrow-derived MSCs (1 × 10^6^) were co-cultured in direct contact with NP cells for 7 days before these activated NP cells were transplanted into the degenerated disc adjacent to the interbody fusion segment. JOA scores were noted to have improved from 14.2 to 27.2 after 36 months, with a resolution of symptoms in all 9 patients involved in the study [51]. When disc hydration was measured with MRI apparent diffusion coefficient (ADC), 67% of cases transplanted with activated NP cells showed no significant radiographic differences at either pre- or post-injection timepoints of 2 and 3 years. These findings suggest clinical improvement occurs independent of radiographic changes within the disc. It also highlights a mechanistic difference, whereby MSCs are used to activate the transplanted NP cells as opposed to other studies, which directly inject MSCs into the disc. Elabd et al. (2016) utilized autologous bone marrow-derived MSCs (15–51.6 × 10^6^ cells) cultured in hypoxic conditions from a cohort of five patients with chronic back pain and injected them into the involved degenerated disc (all were L5–S1). They reported improvement in overall quality of life, strength, and mobility after 48–72 months [49]. In this non-randomized study, non-validated outcomes were used as endpoints, and the disc height in all five patients was either maintained or mildly worsened. The reported clinical and radiographic effects occurred independently of cell dose. A separate non-randomized, prospective study injected autologous MSCs of different doses (mean 2.3 × 10^6^, range 1.72–4.5 × 10^6^) into the discs of 33 patients with low back pain and degenerative disc disease [48]. Using the NPS outcome measure, a mean NPS change of 2.3 over 38.9 months was observed. This finding represented a 55% reduction in pain, which was maintained from 3 months post-treatment. For patients with reported FRI outcomes (N = 6), the average change in score from the baseline (FRI change score) was 4.9 at 12 months to 35 at 5 years. The earliest reported improvement was noted at 3 months. It is important to note, the minimal clinically important difference for FRI is 8.4 points, which was not met by this study [56]. A total of 66% of the discs injected were L5–S1. Twenty of the treated patients underwent post-treatment MRIs and 85% of these had a reduction in the disc bulge size, with an average reduction size of 23% post-treatment. The only significant correlation between the disc bulge reduction and pain improvement was noted for 8 of the 20 patients, who reported >25% disc bulge reduction at 6 months with associated lower NPS scores (*p* < 0.05). These findings support earlier work suggesting a clinical benefit from disc injections of autologous bone marrow-derived MSCs. This benefit is independent of MSC dose and does not correlate significantly with the disc hydration content post-injection. These studies are limited in that they are non-randomized, single-arm, cohort studies with no control group.

In a prospective, non-randomized cohort study, Pettine et al. used autologous MSCs (121 × 10^6^) in 26 patients with indications of spinal fusion or disc replacement [56]. They reported an improvement of between 66% and 56% in VAS and ODI, as early as 3 months post-treatment. These outcomes were sustained through 36 months. Further, 6 of the 26 patients required surgical treatment to improve symptoms within the 3-year study period. Half of the patients received the 1-level injection and the other half 2-levels. The majority (53%) were injected at L5–S1 with 46 % (18/39) at the level above (L4–5). When higher cell concentrations were injected into the discs, they reported a significant effect on the ODI and VAS scores. Specifically, higher concentrations of cells led to improvements of at least 1 grade in the modified Pfirrmann score in 40% of patients (8/20) over 12 months. Moreover, 50% of patients showed an improvement of at least one Pfirrmann grade for 1-level injections versus 30% for 2-level injections.

**Figure 4:**
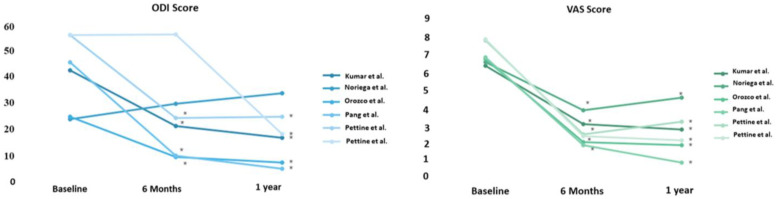
ODI and VAS score changes at baseline, 6 months, and 1 year. * Statistically significant results. ODI—Oswestry Disability Index (0–100, 0 = no disability, 100 = maximum disability possible), VAS—visual analog scale for pain (0–10, 0 = no pain, 10 = worst possible pain), [46,50,52,53,54,55].

While some clinical evidence suggests a potential role for MSCs to affect VAS and ODI in patients with back pain (Figure 4), the exact mechanisms remain elusive. Animal studies have shown that MSCs injected into the nucleus pulposus are able to survive and proliferate [57], and potentially improve the condition of the degenerated discs [58]. These studies raised the intriguing question of the potential mechanisms for therapeutic effect since only a fraction of patients received any benefit. One thought is that the MSCs differentiate into cells that express either a chondrocyte-like phenotype, or an NP cell-like phenotype and that this potentiates the restoration of proteoglycan synthesis [59]. This notion is suggested by the increased production of glycosaminoglycans and keratin sulfate proteoglycans in MSC-transplanted discs in a rabbit model [59]. Another proposed mechanism involves MSCs providing a regenerative environment for residual NP cells in the degenerated disc. This idea is supported by animal studies, which show that cell proliferation, DNA synthesis, and proteoglycan synthesis of NP cells are significantly upregulated after cell-to-cell contact when cocultured with MSCs [60]. It also provides a potential mechanistic basis for studies by Mochida et al. where MSCs were cocultured with NP cells prior to transplanting activated NP cells into degenerated discs [51]. The culture medium also demonstrated increased levels of growth factors associated with the cellular proliferation of NP cells. Altogether, these represent preliminary in vitro and animal studies, yet further research in humans is essential.

Two main strategies have been employed for MSC-based therapies for back pain associated with degenerative disc disease: transplantation of undifferentiated stem cells in vivo with induction under the stimulation of local factors, or differentiation of stem cells in vitro prior to transplantation. In either case, modest improvement was noted in the reviewed studies (Table 1). Significant limitations included the fact that these are non-randomized, single-arm, underpowered studies without the appropriate control groups. Moreover, in the absence of a control arm, the noted increased improvement with a higher dose of cells could be attributed to the placebo effect or to an increased local inflammatory response. Disc hydration was also observed in one report to correlate with an improved pain and function status [46]. However, the disc hydration status did not change significantly at 6 months (1% increase), although it increased by 9% at 12 months (*p* > 0.05). Given that the most clinical benefit (68% reduction in VAS, 60% reduction in ODI) was noted to occur within the first 3 months, it is difficult to fully attribute this effect to the degree of disc hydration, which becomes statistically different at 12 months. In addition, the absence of the appropriate control arms limits the ability to interpret this finding because not all the patients with improved disc hydration status benefited from the MSC injections. Further studies exploring the safety, efficacy, and long-term implications in different populations are warranted. Additionally, future studies will need to investigate the potential mechanisms of MSCs and their ability to induce local cellular proliferation in vivo or prevent further disc degradation by providing a better microenvironment for cell viability. Lastly, the biomechanical properties of the discs treated with MSCs need to be explored further to better understand the benefit of applying this approach in humans.

### 3.4. Clinical Trials: Current and Emerging Cell Therapies for Intervertebral Degenerative Disc Disease

To date, there is no clinical therapy that successfully targets and reverses disc degeneration. Treatment options for discogenic back pain involve non-surgical approaches or surgery for symptomatic patients requiring decompression of their neural elements and stabilization spinal stabilization. With the advent of cell-based strategies and new areas of investigation focusing on regeneration, significant advances have been made, yet more questions arise regarding the therapeutic advantage of stem cells.

MSCs have been identified as being capable of potentially providing the ideal treatment for degenerative disc disease [29,46,47]. Two main strategies exist for using MSCs in treating degenerative disc disease. The first employs transplanted undifferentiated stem cells in vivo, and then, induces them to undergo differentiation under the stimulation of local factors. The other strategy uses stem cells in vitro, where they are differentiated prior to transplantation. Early work with animal model studies provided key investigative tools and the foundation for stem cell use, with promising findings demonstrating the ability of stem cells to differentiate into NP or AF cells and promote extracellular matrix synthesis [57,59,61,62,63,64,65]. These studies made use of coculture systems with growth factors or models, whereby MSCs are injected directly into the disc. The clinical relevance of the animal model data was revealed in the findings that intradiscal injection can lead to ECM synthesis and restoration of disc height [57,59,61,62,63,64,65]. Pooled analysis of several animal stem cell studies for disc degeneration showed significant improvement in both objective imaging findings (disc height index and magnetic resonance imaging T2 signal) and on the cellular level in increased expression of type II collagen and improvement in histological degenerative grading [66]. Moreover, MSCs demonstrated the ability to have anti-inflammatory properties in downregulating proinflammatory cytokines that are known to contribute to discogenic back pain [45]. Altogether, irrespective of the stem cell treatment strategy, the ideal modality would conserve the physiologic function and environment of discs, while reversing the degenerative cascade that ensues as a function of age and other contributing factors. Table 1 summarizes some of the important clinical findings for disc regeneration.

Clinical trials utilizing intradiscal injection of mesenchymal stromal cells (MSCs) from adipose tissue or bone marrow have successfully yielded promising results in patients with discogenic back pain. Emerging trends, as noted in several clinical trials using an intradiscal injection of bone marrow (BM)-MSCs, demonstrated significant pain relief with improvement approaching 71% of optimal during the first year [38,39,40]. In addition, patient mobility and quality of life were noted to have been improved for up to six years post-treatment. While disc rehydration was noted in this cohort, disc height restoration was not observed [40]. This certainly raises the intriguing question of the role of disc integrity and composition versus height restoration for symptom relief. Moreover, higher doses of MSCs improved patient outcomes with fewer patients requiring surgical intervention [38,41,44]. We reviewed the use of different MSCs strategies in clinical trials and summarized their significance in Table 2.

### 3.5. Allogenic Bone Marrow Mesenchymal Stem Cell

Noriega et al. (2017) conducted a randomized controlled trial of 24 patients to determine if allogenic BM MSCs injected into degenerative IVD decreased pain and improved function. The primary outcome measures were self-reported pain on the visual analog scale, and enhanced function as measured using the Oswestry Disability Index, and Short Form quality of life (SF-12) functional scales. Outcomes were assessed at baseline, 3, 6, and 12 months. Clinical significance was not predefined in this study. MRI was used to assess disc height and water content. Study inclusion required patients to be healthy from a hematological and biochemical standpoint. The patients had low back pain with DDD at 1 or 2 levels and failed conservative measures for at least 6 months. Radiographic measurement of IVDs had decreased disc heights of greater than 20% and MRI Pfirrmann grades of 2 to 4. The indexed grades had enough degeneration to observe a treatment effect while permitting the annulus to hold the implanted cells. Under local anesthesia, the test group (*n* = 12) received intradiscal injections of 25 × 10^6^ cells in 2 mL of saline per disc treated, while the control group (*n* = 12) received a sham infiltration of 2 mL of 1% mepivacaine anesthetic of paravertebral musculature close to the affected disc(s). The mean age of the patients was approximately 38 years and 30% were female. The authors reported the temporal evolution of pain and disability as being significantly improved and used ANOVA for pair populations. There was no significant improvement in SF-12, disc height, or water content by MRI. Yet the Pfirrmann scores improved in the MSC-treated patients when assessing several disc parameters for changes in apparent diffusion coefficient versus T2 quantitation. Upon calculation of the statistical testing, using unpaired t-tests to assess mean differences and corresponding confidence intervals, no differences between the treatment and control groups were noted. No major adverse events were identified in either treatment group. The authors reported allogenic MSC recipients required fewer nonsteroidal anti-inflammatory drugs versus sham (66.6% vs. 25%, respectively), and 8.3% of both groups received opioids.

This study adds to the current literature because it is the only RCT comparing expanded allogenic MSCs from healthy donors used in a treatment group versus a sham-control (local anesthetic into the paravertebral muscle) group. Allogenic MSCs have advantages, including lower costs, possible higher homogeneity, and logistically more convenience for clinical applications. However, the limitations include a small sample size, which may account for the lack of statistical significance between the MSC and sham-control groups for pain, function, quality of life, and MRI disc height assessments.

While these results are not yet published, another novel, allogenic BM MSC study, which used a more purified monoclonal population of stromal precursor antigen (STRO-3+) selected progenitor cells has been investigated [67]. Preclinical studies have demonstrated the ability of such progenitor cells to repair disc tissue and reduce inflammation [58,64]. Moreover, because of the immunoselection, these mesenchymal precursor cells (MPCs) are an improved source of colony-forming unit fibroblasts (CFU-Fs), which have the potential to differentiate into tissues of mesenchymal lineage and an increased potency for self-renewal [62].

The Rexlemestrocel-L phase-3 trial was a multicenter, randomized, double-blind, placebo-controlled study to evaluate the efficacy and safety of a single injection of rexlemestrocel-L alone or in combination with hyaluronic acid in subjects with chronic low back pain. The study enrolled 404 patients with documented DDD, Pfirrmann scores from 3 to 6 at a single level from L1 to S1, and with low back pain for at least 6 months, which was not responsive to conservative treatments, including physical therapy. The patients were randomized to receive 1 × 10^6^ rexlemestrocel-L cells, 1 × 10^6^ rexlemestrocel-L cells with hyaluronic acid (HA), or a saline solution (placebo) control. A primary endpoint was examined for the treatment success based on a composite of 50% or greater pain reduction in the VAS score, a 15-point or greater reduction in the ODI score, and a lack of post-treatment interventions at 12- and 24-months post-injection. These specific results and the analysis were not reported in the publication. Only the demographics, mortality, and serious and other adverse events were reported on clinicaltrials.gov. There were no deaths due to any cause during the clinical study. The percentage of serious adverse events in the group receiving 1 × 10^6^ rexlemestrocel-L cells alone (*n* = 143), 1 × 10^6^ rexlemestrocel-L cells with hyaluronic acid (*n* = 129), and saline placebo (*n* = 132) were 9.29%, 9.38%, and 5.38%, respectively. Other non-serious adverse events were reported among all three groups, including musculoskeletal and nervous system disorders, such as back pain, muscle spasms, and hypoesthesia or paresthesia. The outcome measures proposed for the analysis were similar to the Noriega study, in terms of assessing the VAS and ODI. In contrast, the rexlemestrocel-L study defined success based on composite responder analyses, which included the reduction in pain (VAS), improvement in function (ODI), and lack of post-treatment intervention (decrease in the need for another treatment for LBP).

Similar to the Noriega study, cells were isolated from bone marrow and from mononuclear cells and expanded ex vivo. However, the rexlemestrocel-L cells originated from a single, young healthy donor, while in the Noriega study, they were isolated from four men and one woman, while the cells obtained from each donor were used for one to three recipients. In all cases, the cell products were examined for the presence of donor infectious diseases and screened for viability before use.

Two other investigations recruited patients to assess the efficacy of intradiscal injection of autologous bone marrow-MSCs. The DREAM trial is assessing intradiscal treatment injections in subjects with chronic LBP of >6 months, who have up to 3 levels of intervertebral disc degeneration (IVDD) and are unresponsive to conventional therapies [68]. The ACTIVE trial will assess similar intradiscal treatment injections in worker patients, using nearly identical criteria apart from including up to 4 levels of IVDD [69]. This is an important distinction since occupational incidence rates of LBP can vary and may be attributed to missed work and longer-term disability. These are phase II B efficacy, single-center, randomized, and controlled double-blinded trials. The treatment groups will have bone marrow harvested from the iliac crest and receive single injections of 1.5 × 10^7^ autologous BM-MSCs to each affected disc. The sham comparators will have simulated bone marrow harvesting without insertion into the iliac crest and simulated local anesthetic without a disc or placebo injection. For the DREAM trial, primary outcome measures include pain relief as measured by a decrease in VAS and functionality by ODI. Secondary measures will assess the quality of life (SF36) and its evolution, interval disability and pain evolution, employment status, and pain medication consumption. For the ACTIVE occupational study, efficacy will be evaluated 12 months after treatment in terms of pain relief (VAS), function (ODI), quality of life (SF36), and work ability index (WAI).

### 3.6. Allogenic Discogenic Cells

A unique clinical trial that is currently active involves the application of a novel cell population produced by expanding and modifying cells from adult intervertebral nucleus pulposus tissue [70]. Since the cells are of intervertebral disc origin, they are called discogenic cells, yet they have a different phenotype than the original disc-derived cells because of the culture environment used for the in vitro expansion. Preclinical in vitro and in vivo models have demonstrated that the cells are non-tumorigenic, exhibit multipotentiality for mesenchymal lineage, and can produce an extracellular matrix, which may rebuild depleting tissue within degenerating discs [41,62].

There is evidence that nucleus pulposus stem cells isolated from degenerated IVD have demonstrated impaired proficiency in proliferation and differentiation. When cultured in a simulated disc microenvironment, such cells are more resistant to hypoxic and acidic pH compared to other types of stem cells, such as adipose stem cells [62,71]. The potential for nucleus pulposus stem cells leading to IVD regeneration makes it desirable for further investigation [62]. No current cell treatments in human clinical research to treat DDD have demonstrated IVD regeneration, although improvement in pain and function have been reported. This may be due to the stem cell potential for paracrine activity influencing anti-inflammatory properties. Some of the earlier referenced studies that denoted improvement in MRI radiographic changes in the water content of the disc support the idea that cell therapy has the potential to reverse degenerative cascades. However, more investigations are needed given the lack of documented, specific standardization in the type of MRI assessment, and overall heterogeneity and variability in the studies.

### 3.7. Autologous Bone Marrow Mesenchymal Stem Cells

A new clinical trial will be conducted to assess the percutaneous fluoroscopic delivery of autologous BM MSCs for the treatment of symptomatic degenerated intervertebral disc disease [68]. A total of 24 participants will be equally randomized into two groups: 12 healthy control subjects and 12 treatment subjects. Then, the treatment group will be subdivided and randomized into 6 subjects receiving a low dosage treatment of 1–2 mL of a 2 × 10^6^/mL concentration solution of MSCs and 6 subjects receiving a high dosage treatment of 1–2 mL of a 4 × 10^6^/mL concentration solution of MSCs. Primary outcome measures will be of TRAEs and SAEs, while secondary outcome measures include changes in VAS, ODI, SF-36, and changes in MRI monitoring of the transplant site. Inclusion criteria include the absence of infection and coagulopathy, symptoms despite conservative (non-surgical) management for >6 months, absence of facet joint pain involvement between 18 months and 2 weeks, confirmed by diagnostic medial branch block or facet joint injections, and a Pfirrmann score of 3 to 5. Some exclusion criteria include infection, cancer, severe spinal stenosis, pregnancy, contraindications to MRI and immunosuppression, and a BMI of >35. This study will add to the literature as a comparison between autologous BM-MSCs and allogenic MSC studies, using relatively similar study subjects. It will also provide insight into lessons learned from autologous preparation, handling, and delivery. Previous investigations have demonstrated morbidities such as chronic pain from the use of autologous iliac crest bone grafts, as well as limited quantity and quality dependent on the age and comorbid diseases of the patients.

### 3.8. Adipose Mesenchymal Stem Cells

Kumar et al. (2017) investigated the intradiscal injection of hyaluronic acid derivative and adipose-tissue-derived expanded MSCs from abdominal fat in patients with discogenic LBP [50,72]. The primary endpoints were the safety and tolerability of the combined implantations of AT-MSCs and HA derivatives in patients with chronic LBP. Systemic monitoring included physical and neurological examinations, monitoring of vital signs, and peripheral blood testing. Ten patients had chronic (>3-month) axial discogenic LBP, with a mean age of 43.5, while four female patients who failed conventional treatment had no prior surgery. The levels treated in this case series included 90% L4–5 (9/10) patients and 10% L5–S1 (1/10) patients. Five patients received injections of AT-MSCs at a dose of 2 × 10^7^ and HA and another five received a higher dose of 4 × 10^7^ cells/disc. No control group was available for comparison. Outcome measures included SAEs, VAS, ODI SF-36, and MRI Pfirrmann ADC mapping to assess DD severity as well as an X-ray for disc height. There were reported improvements in VAS and ODI over time from baseline, 3-month, 6-month, and 12-month assessments. There was no significant difference between the groups receiving different doses at 12 months. SF-36 was not reported. One patient had an MRI Pfirrmann improvement from 4 to 3. Three patients showed increased water content through MSC application and no decrease in height on an X-ray. No observation of AEs or SAEs was related to the cell transplantation and abnormal blood tests were assessed during the first week and quarterly.

Comella et al. (2017) examined the effects of intradiscal implantation of stromal vascular fraction (SVF) of adipose-derived MSCs plus platelet-rich plasma (PRP) in patients with degenerative diseases [73]. While the regenerative potential of MSCs was recognized, it is the paracrine activity of MSCs that is believed to be a primary means through which such cells mediate their anti-inflammatory, anti-apoptotic, angiogenic, and restorative properties. Cell signaling and complex interaction of biological mediators secreted by MSCs help regulate the regeneration of damaged body tissues. The precursor to MSCs is the pericyte, which are cells present on microvessels and capillaries and in tunica adventitia around larger vessels. Hence, SVF may confer enhanced regenerative potential when combined with PRP, which can secrete growth factors to supplement the SVF effects. In this study, patients with DDD of 1–3 lumbar discs with axial LBP after conservative medical and physical therapy for over 6 months were eligible [73]. An intact annulus fibrosus capable of holding the cell implantation, as demonstrated by MRI, was required. Subjects with congenital or acquired spinal deformities, active cancers or infections, spinal instability, stenosis, and more than 50%-disc height loss or Modic III changes on MRI were excluded. The primary safety outcome was the incidence of SAEs over 6 months. Other outcomes included VAS, present pain index (PPI), ODI, SF-12, the Beck Depression Inventory (BDI), and other functional and psychological questionnaires. A total of 60 mL of abdominal fat was collected using an aspiration cannula and the tissue was prepared using an adipose stromal vascular fraction preparation kit. After digestion with collagenase, it was centrifuged, washed twice, and filtered to remove any enzymes, before the SVF was resuspended in 1–3 mL of autologous PRP. Fluoroscopy-guided injection of the treatment was performed immediately after a small amount of contrast agent was injected. Approximately 30–60 million SVF cells were obtained. No severe adverse events (SAEs) were reported during a 12-month follow-up period with no incidences of infection. Patients demonstrated improvements in multiple parameters, including flexion, pain ratings, VAS, PPI, and short-form questionnaires. The study enhanced our understanding of AD MSCs, whereby treatment as part of SVF can be feasible without in vitro culture expansion, pretreatment collection, preparation, and delivery after fluoroscopic confirmation of indexed IVD can occur. This implies a “one-step” approach for treating patients with PRP-activated cell therapy and bypassing a need for culture expansion. The study was limited by a small sample size (*n* = 15) with no comparison group, which makes it difficult to draw a conclusion that the effects observed are reflective of true effects. Future studies and larger, placebo-controlled investigations will be beneficial.

A new clinical trial will assess the safety and efficacy of Matrilin-3 pretreated autologous AD MSC implantation. Matrilin-3 is an extracellular matrix (ECM) protein involved in cartilage development. The role of ECM components in the remodeling process of disc degeneration has garnered much attention as manipulation of these elements may provide adjunctive therapy for improving disease outcomes [50]. Similarly, investigations into extracellular vesicles (EVs) as active biological substances for intercellular communication with the ability to promote tissue regeneration have gained popularity [74]. These strategies consider a need to address the challenging microenvironment of a degenerative IVD for repair and regenerative in stem cell investigations.

Targeting ECM remodeling as an adjunct therapy may result in better disease outcomes. Inclusion criteria include an age of 19–70 years, ODI score ≥ 30%, VAS ≥ 4, MRI Pfirrmann classification 3–4 between lumbar-1 and sacral-1, and the confirmation of one or two degenerated discs, which have been confirmed by injection procedures [75]. While the details of the various injections confirming the diagnosis of LBP are provided, it is worth noting the perceived value of using discography as a prerequisite to identify symptomatic degenerated discs in stem cell study designs. While some may suspect this as a confirmatory aid in the diagnosis, injecting AD MSCs at the time of provocation discography results in a painful response to the indexed disc from pressurization upon further contrast injections. It follows that a variable volume of AD MSCs may be introduced based on patient tolerance. For this reason, not performing discography may allow the introduction of the cellular product and evaluation of the proposed cell delivery. Results of studies that include discography will add value in a way that we are able to discern potential benefits or limitations to the immediate use of discography as a means for the treatment of DDD.

### 3.9. Human Umbilical Cord Mesenchymal Stem Cells

A clinical trial is actively recruiting to evaluate the safety and effectiveness of human umbilical cord mesenchymal stem cells (hUC-MSCs) for the treatment of lumbar DDD [76]. This phase 1 trial will enroll twenty participants with lumbar DDD who are diagnosed with lumbar disc herniation and operated on with full endoscopic lumbar discectomy. The patients will receive twenty million hUC-MSCs, which will be immediately injected into the degenerative discs. The primary outcome measure includes changes in lumbar disc MRI signaling values from the baseline through 3, 6, and 12 months. Secondary outcomes include VAS, ODI, SF36, the disc height index from an X-ray, and the side of the herniated nucleus pulposus (HNP) from an MRI. The participants will also be monitored and assessed for TEAEs and SAEs for up to 12 months. Preclinical animal studies have demonstrated the ability of hUBC MSCs to survive and undertake a chondrocyte-like phenotype when injected into the IVD [77,78].

### 3.10. Adult Juvenile Chondrocytes

Intradiscal injection of expanded allogenic juvenile chondrocyte cells (density of 5 × 10^7^ cells/mL) for refractory LBP was investigated [79]. Coric et al. (2013) studied the effect of allogenic juvenile chondrocytes as a cell-based biologic for disc repair by assessing clinical and radiographic outcomes at 12 months [80]. Fifteen patients with single-level DDD, LBP greater than or equal to three months who failed conservative with no prior surgery were included, while no comparison group was available for this cohort investigation. Outcome measures were pain NRS, ODI, physical SF-36, and MRI. The ODI, NRS, and SF-36 (*p* = 0.0002) all improved significantly from the baseline and 10/13 patients had an improved MRI. HIZ was absent or improved in eight patients. The lack of a comparison group limits the quality of evidence. The investigation elucidates the use of chondrocytes, which synthesis and turnover a large volume of the extracellular matrix (ECM) in treating DDD. Breakdown of ECM and loss of proteoglycans and type II collagen are observed in the DDD.

### 3.11. Autologous Disc Derived Chondrocytes

Novocart^®^ disc plus (ND plus) is a biologic-device combination product (combination of autologous disc chondrocyte and a self-polymerizing hydrogel) investigated for the treatment of lumbar DDD. ND basic is the device alone with no active chondrocyte cells. Tschugg et al. (2017) examined whether autologous disc chondrocyte transplantation is safe and feasible [81]. Twenty-four patients with single-level herniation, no previous surgery, no extensive annulus damage, and no other disease by MRI who failed conservative or interventional treatment for DDD were included. All received sequestrectomy as part of the treatment. The mean age was 42 and 10 patients were female. Twelve patients were randomized to receive ND plus, while the other twelve received ND basic. Four of the patients who received ND basic dropped out of the study. The biopsies taken during the discectomy removed a region of the herniated disc. The chondrocytes were isolated, expanded, and suspended into the hydrogel for implantation via injection into the nucleus pulpous. Safety outcomes included measurement of ESR, CRP, and changes in blood pressure, pulse rate, and MRI assessment. No predefined clinical significance parameters were indicated. Adverse effects were more common in the ND plus group (50% 6/12) compared to the ND basic (25% 2/8). One treatment event was considered serious: IVD protrusion and the patient underwent surgery. One patient experienced spinal pain 21 days post-implant. Nasopharyngitis was the most common event deemed unrelated to the transplantation. This investigation adds to the literature because it suggests the possibility of taking autologous discs from the patient’s own IVD for transplantation. This may hold advantages because endogenous progenitor cells, as a cell source, may respond in a more favorable manner to the special microenvironment of the IVD, under degenerative conditions, compared to other stem cell sources.

### 3.12. Other Clinical Investigations

Five of the research studies referenced were either withdrawn or terminated from current and emerging clinical trials. These included two autologous BM MSCs, two adipose-derived MSCs and an allogenic MSC study to be used with lumbar fusion [82,83,84,85]. The reasons for withdrawal or termination included a lack of funding, an unwillingness to continue the clinical trial, a transition of the principal investigator, and the preference for a better study design. One investigation on the autologous BM MSCs was suspended due to awaiting a sponsor and FDA feedback prior to its resumption [86]. Three more investigations on autologous adipose MSCs, autograft tissue MSCs, and allogenic umbilical cord MSCs had unknown statuses [69,72,87]. This means that their recruitment statuses have not been updated in 2 years and the study completion date has elapsed.

One clinical trial was performed to isolate and authenticate MSC-like progenitor cells from degenerated IVDs from the lumbar spine [88]. While no published data were available from this trial, the evaluation is significant because investigations suggest that native IVD cells may be better able to tolerate the acidic and hypoxic microenvironmental conditions of the degenerative disc [89,90]. This use of such discogenic cells as progenitor cells in the treatment of DDD is currently under investigation [70].

## 4. Conclusions

Stem cell-based therapies that integrate tissue-engineering technologies and biomaterials science are becoming fundamental pillars in regenerative medicine. Clinical success with animal model studies employing stem cell transplantation is promising and provides a good foundation for further studies on stem cell transplantation strategies. Ongoing clinical trials will be crucial in validating the results in humans and serve as a launch pad for further stem cell therapeutic strategies. However, the clinical outcomes of stem cell transplantation for specific symptoms, such as back pain, as well as other back-related disorders, while encouraging, remains poorly understood and warrant further studies.

The use of stem cells for regenerative therapy has provided insight into potential therapeutic options for discogenic back pain. While cell-based strategies hold significant opportunity for regenerative medicine, its role in treating degenerative disc disease associated with discogenic back pain is still in the early stages of clinical trials and the overall efficacy has yet to be proven. Although stem cell therapy is a rapidly emerging field given its perceived potential, it should be emphasized that these preliminary research findings need to be approached with great caution from a patient’s perspective. Specifically, the Federal Drug Association (FDA) has not sanctioned or approved the use of stem cells for degenerative disc disease or associated discogenic back pain. On the contrary, the FDA has issued warnings and raised concerns about scams by clinics and companies marketing stem cell treatment without FDA approval. Undoubtedly, further randomized, long-term clinical studies are required to establish the full potential of stem cell regenerative therapy. Upon FDA approval, understanding how stem cells might be integrated into the current treatment guidelines and management strategies for patients with discogenic back pain.

From a clinical perspective, being able to reliably identify candidates for intradiscal injection and having an objective means of assessing their improvement will prove challenging given the multifactorial complex nature of back pain. Height restoration of a degenerative disc might serve to effectively monitor or predict improvement, although it would have its limitations in that pain relief has been observed in patients receiving intradiscal stem cell injection with no disc height restoration. Thus, as we already know, not all back pain is discogenic in nature and the perceived potential mechanism for stem cell therapy would be for back pain associated with degenerative disc disease. Establishing non-invasive means for selecting patients that might benefit from stem cell injections will be paramount. Moreover, the long-term implications of stem cell transplantation with respect to safety and efficacy need to be better defined prior to making this tissue regenerative approach a standard of care. This underscores the need for future studies to focus on being able to evaluate the safety and efficacy of stem cell treatment when compared to current non-surgical treatment standards and perceived long-term outcomes.

## Figures and Tables

**Table 1:** Intradiscal Mesenchymal Stem Cell Injections for Discogenic Lumbar Back Pain in Humans.

Study	Sample Size/ Study Design	Patient Demographics	Follow up (Months)	Involved Levels	Dose Regimen	Main Results
Centeno et al. [48]	N = 33 Cohort	Age: 19–72 yo Mean 40.3 yo 21 M 12 F BMI: 24.8	72	All single-level injections L5–S1 (*n* = 22) L4–L5 (*n* = 11)	Single injection Autologous 2.3 × 10^7^ cells/disc range 1.73–45 × 10^6^	Mean NPS outcome scores: •At baseline—5.2, 3 months—1.6 *, 12 months—0.6 *, 24 months—1.3 *, 48 months—2.5 *, 6 years—3.7 Mean FRI outcome scores: •At baseline—61, 3 months—20 *, 12 months—4.9 *, 24 months—17 *, 48 months—19 *, 6 years—30
Elabd et al. [49]	N = 5 Nonrandomized cohort with no control group or validated endpoints	Age: 25–53 yo 3 M 2 F	48–72	5 injections at L5–S1 (1 patient received two injections 1 year apart)	Single injection Autologous bone marrow-derived MSCs 15.8–37.3 × 10^6^ cells/ disc hypoxic cultured	•No adverse events related to MSC injection •All patients maintained or mildly worsened disc height •Majority reported an overall improvement in QoL, strength, and mobility. •Effect independent of cell dose
Kumar et al. [50]	N = 10 Single center, single arm, open-label phase 1 clinical trial	Age 30–64 yo Mean 43.5 yo 6 M 4 F BMI 20.2–30.8	12		Autologous MSCs isolated from subcutaneous. abdominal adipose tissue. Number of cells: 4.7–5.4 × 10^7^	Mean VAS scores: •At baseline—6.5, 1 month—4.6 *, 3 months—4.3 *, 6 months—4.3 *, 12 months—2.9 * ODI scores: •At baseline—42.8, 1 month—31.2 *, 3 months—31.7 *, 6 months—21.3 *, 12 months—16.8 * SF-36: No difference between the groups at 12 months (significant improvement in 6/12 patients).
Mochida et al. [51]	N = 9 Single-arm cohort with no control group	Age: 20–29 yo 8 M 1 F	36	L5–S1 (*n* = 4) L4–L5 (*n* = 4) 2 patients with lumbar disc herniation; 7 patients w/ lumbar discopathy.	1 million activated autologous NPcells suspended with sterile saline were transplanted into the degenerated disc adjacent to the fusion level 7 d after the first fusion surgery	Mean JOA Score: •At baseline—14.2 •36 months—27.2 •No injected disc showed worsening degeneration on MRI at 36 months.
Noriega et al. [52]	N = 24 Cohort study with control group	Age: 18–75 yo Inclusion Criteria (Mean 38)	12	The patients had 1 or 2 affected discs, with the lesion located at L1–L2 (*n* = 1), L2–L3 (1), L3–L4 (3), L4–L5 (18), or L5–S1 (15).	Bone marrow was obtained from 5 healthy donors and processed using GMP conditions. Immune matching was not attempted	Lumbar pain VAS and disability (ODI) were reduced at 3 months, 6 months, and 12 months * ODI scores: •At baseline—34 (vs. 24 for control group), 6 month—16 (vs. 30), 12 months—22 (vs. 34) Mean VAS scores: •At baseline—67 (vs. 62 for control group), 6 month—40 (vs. 51), 12 months—47 (vs. 47) •Treated patients had decrease in Pfirrmann disc degeneration grade at 12 months, whereas control group had increase in grade at 12 months *
Orozco et al. [46]	N = 10 Cohort single arm study no control group	Age: 18–65 yo Inclusion Criteria (Mean 35) 4 M6 F	12	L4–L5 (2), L5–S1 (6), or both discs (2).	Autologous MSC transplantation bone marrow volume, 89 ± 5 mL; Expansion was performed under GMP conditions	Lumbar pain VAS and disability (ODI) decreased at 3 months, 6 months, and 12 months * Mean VAS scores: •At baseline—68.9, 6 months—21.6 *, 12 months—20* ODI scores: •At baseline—25, 6 months—9.4 *, 12 months—7.4 * Disc hydration (water content ratios) on imaging improved at 12 months *
Pang et al. [53]	N = 2 Cohort No control group	38 yo M 45 yo F	24	L4–L5 (*n* = 1) L3–L4 (*n* = 1)	Human umbilical cord tissue-derived mesenchymal stem cells (HUC-MSCs) contain stem cells	•Improvement in back pain and lumbar function at 24 months for both patients. •Water content higher in disc of 1 patient at 24 months. T2-change increased at disc level compared to preinjection Mean VAS scores: •At baseline—7, 6 months—2 *, 12 months—1 * ODI scores: •At baseline—46, 6 months—10 *, 12 months—5 *
Pettine et al. [54]	N = 26 Prospective, nonrandomized two-arm study (one vs. two-level injection) at a single center with no control group	Age 18–61 yo Mean 40 11 M 15 F BMI Mean 27, range 19–37) 13 had traumatic cause of injury	12	1-level N = 13 2-level N = 13 L4–L5 level N = 18 L5–S1 N = 21	Autologous bone marrow concentrate (BMC) disc injections.	•At 3 months and 6 months, ODI and VAS scores improved more in higher concentration MSC injection patients versus lower concentration * •Improvement of at least 1 grade in Modified Pfirrmann Score in 8 of 20 at 12 months Mean VAS scores: •At baseline—80.1, 12 months—22.9 * ODI scores: •At baseline—56.5, 12 months—18.3 *
Pettine et al. [55]	Same cohort as above	Same cohort as above	24			Reduction in ODI and VAS scores endured at 24 months *
Pettine et al. [47]	Same cohort as above	Same cohort as above	36			Reduction in ODI and VAS scores endured at 36 months *

* Statistically significant results. FRI—functional rating index (0–100, 0 = functionally independent, 100 = severely disabled), JOA—Japanese Orthopedic Association score (0–17, 0 = severely disabled, 17 = no disability), MRI—magnetic resonance imaging, NPS—numeric pain score (0–10, 0 = no pain, 10 = worst possible pain), ODI—Oswestry Disability Index (0–100, 0 = no disability, 100 = maximum disability possible), QoL—quality of life, VAS—visual analog scale for pain (0–10, 0 = no pain, 10 = worst possible pain). M—male, F—female, yo—years old.

**Table 2:** Clinical Trials: Cell Therapy for Intervertebral Degenerative Disc Disease.

Cell Type	Clinical Trial ID	Phase	Author/Sponsor	Title
Allogenic BM MSC	NCT02412735	Phase 3	Mesoblast, Ltd.	Placebo-controlled Study to Evaluate Rexlemestrocel-L Alone or Combined with Hyaluronic Acid in Subjects With Chronic Low Back Pain
Allogenic BM MSC	NCT01860417	Phase 2	David C Noriega, M.D.	Treatment of Degenerative Disc Disease with Allogenic Mesenchymal Stem Cells (MSV)
Autologous BM MSC	NCT05066334	Phase 2	Campus Bio-Medico University	Efficacy of Intradiscal Injection of Autologous BM-MSC in Subjects with Chronic LBP Due to Multilevel Lumbar IDD (DREAM)
Autologous BM MSC	NCT04759105	Phase 2	Campus BioMedico University	Efficacy of Intradiscal Injection of Autologous BM-MSC in Worker Patients Affected by Chronic LBP Due to Multilevel IDD (ACTIVE)
Allogenic Discogenic Cells	NCT03347708	Phase 1	DiscGenics, Inc.	Study to Evaluate the Safety and Preliminary Efficacy of IDCT, a Treatment for Symptomatic Lumbar Intervertebral Disc Degeneration
Adipose MSC	NCT02097862	Phase 1	Kristin Comella, Ph.D.	Adipose Cells for Degenerative Disc Disease
Autologous Adipose MSC	NCT02338271	Phase 1/2	Hermant Kumar, Ph.D.	Autologous Adipose-Derived Stem Cell Therapy for Intervertebral Disc Degeneration
Autologous Adipose MSC	NCT05011474	Phase 1/2	Inbo Han	Safety and Efficacy Study of Matrilin-3 Pretreated Autologous Adipose Derived Mesenchymal Stem Cells Implantation in Chronic Low Back Pain Patients with Lumbar Intervertebral Disc Degeneration (MANT3_ASC
Human Umbilical MSC	NCT04414592	Phase 1	Shanghai General Hospital	Human Umbilical Cord Mesenchymal Stem Cells for the Treatment of Lumbar Disc Degeneration Disease
Allogenic Juvenile Chondrocytes	NCT01771471	Phase 2	Domagoj Coric, M.D., ISTO Technologies, Inc.	A Study Comparing the Safety and Effectiveness of Cartilage Cell Injected into the Lumbar Disc as Compared to a Placebo
Autologous Disc Derived Chondrocytes	NCT01640457	Phase 1/2	Anja Tschugg M.D., Tetec AG	A Prospective Randomized Multicentre Phase I/II Clinical Trial to Evaluate Safety and Efficacy of NOVOCARTÆ Disc Plus Autologous Disc Chondrocyte Transplantation (ADCT) in the Treatment of Nucleotomized and Degenerative Lumbar Discs to Avoid Secondary Disease

## Data Availability

No new data were created or analyzed in this study. Data sharing not applicable to this article.
